# Emerging HER2 Targeting Immunotherapy for Cholangiocarcinoma

**DOI:** 10.32604/or.2025.065319

**Published:** 2025-08-28

**Authors:** Prin Sungwan, Jutatip Panaampon, Ratchaneewan Sumankan, Genki Aoki, Seiji Okada

**Affiliations:** 1 Division of Hematopoiesis, Joint Research Center for Human Retrovirus Infection & Graduate School of Medical Sciences, Kumamoto University 2-2-1 Honjo, Chuo-ku, Kumamoto, 860-0811, Japan; 2 Division of Hematologic Neoplasia, Department of Medical Oncology, Dana-Farber Cancer Institute, Harvard Medical School, 450 Brookline Avenue, Boston, MA 02215, USA; 3 Department of Biology, Faculty of Science, Chiang Mai University, Chiang Mai, 50200, Thailand

**Keywords:** Cholangiocarcinoma, human epidermal growth factor receptor 2 (HER2), immunotherapy, animal models for cancer study

## Abstract

Cholangiocarcinoma (CCA) is a fatal bile duct malignancy. CCA is intrinsically resistant to standard chemotherapy, responds poorly to it, and has a poor prognosis. Effective treatments for cholangiocarcinoma remain elusive, and a breakthrough in CCA treatment is still awaited. The human epidermal growth factor receptor 2 (HER2) plays an oncogenic role by promoting an aggressive cancer phenotype through multiple pathways. While HER2 has shown increasing potential as an effective target for breast and gastric cancers over the last decade, this has not been the case for CCA. This review explores the possibility of targeting HER2 in CCA immunotherapy. Key findings suggest that HER2 alterations have been reported as one of the signatures associated with a poorer prognosis in liver fluke-associated CCA, the most prevalent subtype in Southeast Asia. Furthermore, we assess recent advances in HER2-targeted therapeutic approaches, presenting the current stage, rationale, and evidence supporting the use of HER2 as a promising therapeutic target for cancer immunotherapy in CCA. We also emphasize the crucial role of animal models in developing anticancer therapies. In summary, focusing on HER2 expression could provide alternative strategies for the HER2-altered CCA cluster.

## 1 Introduction

Cholangiocarcinoma (CCA) is a deadly and aggressive bile duct malignancy [1–3]. Most CCA patients have a poor prognosis with very low 5-year survival rates, and the median survival rate is less than 1 year [4–6]. Moreover, the response rate to standard chemo-drugs is less than 40%. Tumor recurrence and resistant phenotype to first-line therapy often occur [7–9]. There are still no effective treatment regimens or strategies for CCA. Only R0 surgical resection (completely removed, with no microscopic or macroscopic residual tumor at the surgical margins) [10–12] is considered the best strategy for CCA treatment, whilst only a few patients can be operated on with the resection due to a lack of signs/symptoms [10]. The human epidermal growth factor receptor 2 (HER2) is a member of the human epidermal growth factor receptor (HER) family, which plays oncogenic roles in several malignancies. HER2 promotes cancer cell proliferation, differentiation, and survival via multiple signal transduction pathways [13,14]. In the last decade, there has been increasing potential for using HER2 as an effective target for breast and gastric cancers but not in CCA. Recently, the exploration of CCA cancer biology revealed that HER2 alterations have been dominantly observed in liver fluke-associated CCA clusters and are associated with poorer prognosis [15]. Thus, targeting CCA by focusing on HER2 expression might offer alternative strategies for HER2-altered CCA immunotherapy. This review offers the current understanding and rationale for considering HER2 as a novel, promising target for CCA immunotherapy. It also highlights the vital role of mouse models in bridging basic research to clinical studies.

## 2 The Current Status of CCA

Cholangiocarcinoma (CCA) refers to a group of biliary malignant tumors that arise from the bile duct epithelial cells. While CCA is currently considered relatively rare, its incidence is rising globally, representing approximately 3% of all gastrointestinal malignancies [6,16] and 15% [4] of primary liver cancers [8–10]. The worldwide incidence [4,5,9] of CCA is approximately 0.3 to 6 per 100,000 per year [4,8,17]. A notable incidence of CCA has been observed in Southeast Asian countries [5], including Laos, Cambodia, and the northeastern region of Thailand [10], with rates of 17.6 for females and 44.3 for males per 100,000 [8,9,18]. More than 8000 cases were annually diagnosed due to liver fluke infection (*Ophisthorchis viverrini, Opisthorchis felineus* [19], and *Clonorchis sinensis* [8,20,21]). However, CCA has been considered a rare malignancy in Western countries with liver fluke-negative disease (less than 6 per 100,000). CCA could be anatomically classified into intrahepatic CCA (iCCA) (originating above second-order bile ducts) and extrahepatic CCA (eCCA) (arising below second-order bile ducts). eCCA is further divided at the cystic duct into perihilar CCA (pCCA) and distal CCA (dCCA) [4,13]. Most patients diagnosed with CCA are typically in the advanced stages of the disease, as CCA is often asymptomatic in its early phases. Despite the extensive literature available on CCA, effective treatment targets and early diagnostic markers remain inadequately defined. Currently, R0 resection is considered the gold standard for CCA treatment; however, only approximately 3% of patients are eligible for this surgical option [22]. Consequently, more than 90% of CCA patients experience a poor prognosis, with a median overall survival (mOS) of 4 months, a one-year survival rate of 14.1%, and a two-year survival rate of 7.3% [8,9,23]. While many standard chemotherapies such as gemcitabine, 5-fluorouracil (5-FU) [24], cisplatin, and sorafenib [25] have been used as adjuvant therapy, and demonstrated poor clinical outcomes [17,26]. For instance, 5-FU illustrated a poor response rate ranging from 0% to 40% and a median survival rate of approximately 2 to 12 months [8,27,28]. Clinical trials of targeted therapies of unresected and mixed CCA populations revealed minimal response [29–31]. Recently, the TOPAZ-1 trial [32,33] and the global real-world analysis proposed the combination of immune checkpoint inhibitor, durvalumab plus gemcitabine, and cisplatin as a novel first-line standard care for advanced CCA. The combination significantly improves clinical benefits, with an overall response rate (ORR) of 32.7%, with stable disease in 45.2% of patients. Any grade adverse events (AEs) occurred in 92.9% of patients (grade > 2: 46.6%) [34]. Moreover, resistant phenotype to standard chemo-drugs and tumor recurrence after treatment still occurred [35–37].

## 3 The Human Epidermal Growth Factor Receptors 2 (HER2) in Cancers

**Figure 1:**
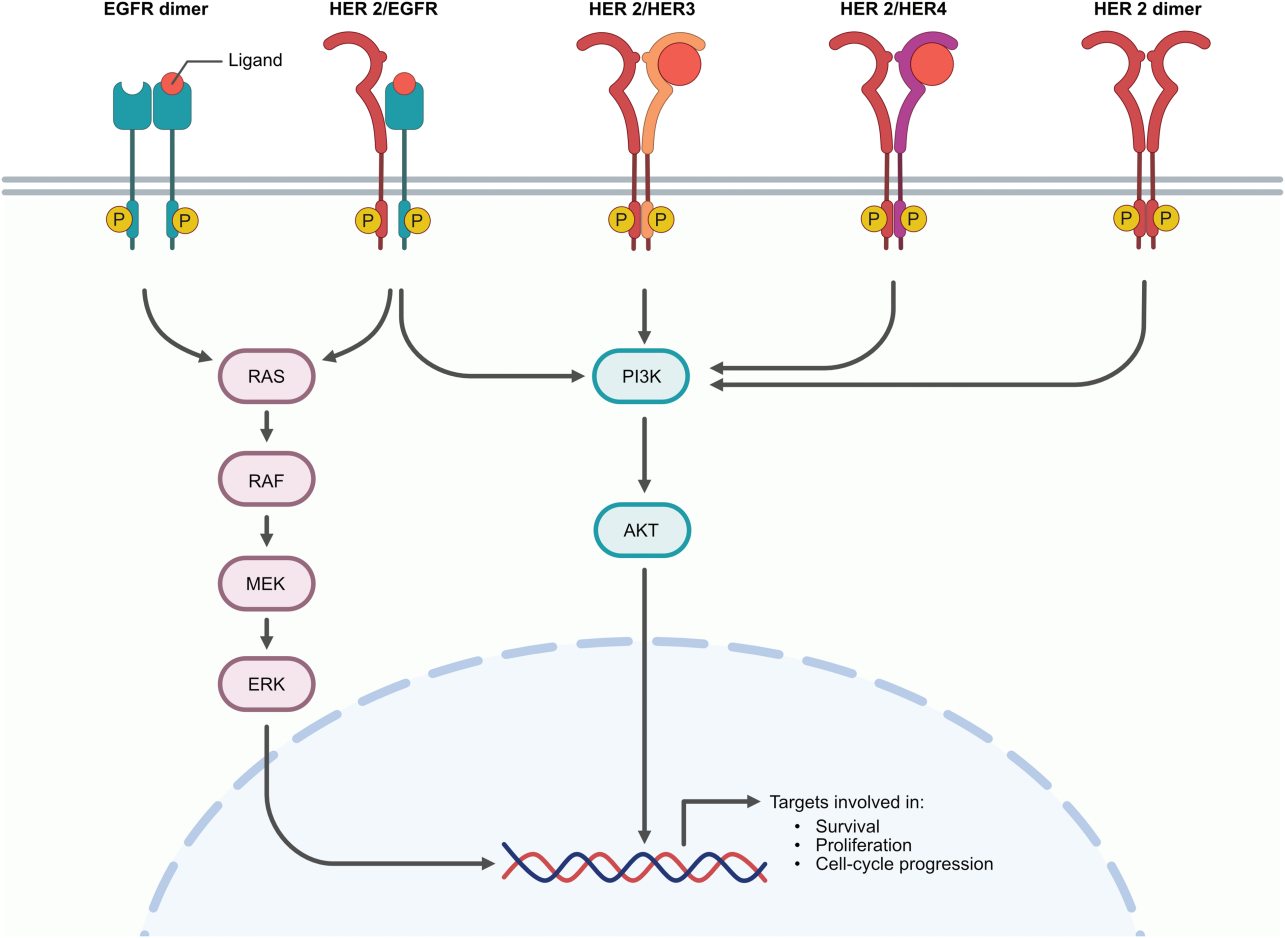
HER2 dimerization with other EGFR; HER2 activation leads to the upregulation of several oncogenic cascades such as PI3K/Akt pathways, RAS/RAF, and MAPK pathways [14,40] (Created with BioRender.com)

HER2 is a member of the HER family, the tyrosine kinase receptor group which plays oncogenic roles in several cancers such as breast, pancreatic, and gastric cancers [38–40]. The family includes HER1 (known as EGFR), HER2 or ErbB2, HER3, and HER4 (Fig. 1) [41,42]. This receptor family comprises four parts, including the ligand-binding region, transmembrane, C-terminal, and cytoplasmic tyrosine kinase domain. The binding of ligands triggers homo- or hetero-dimerization leading to activation of the tyrosine kinase domain by tyrosine-residue phosphorylation. Although HER family members exhibit similar characteristics, HER2 is distinct due to its absence of specific ligands. Consequently, HER2 signaling predominantly depends on homo- or hetero-dimerization (more potent via hetero-dimerization). HER2-HER3 hetero dimerization is the most prevalent and potent oncogenic stimulator [43,44]. HER2 activation results in the upregulation of several oncogenic downstream pathways, including the phosphoinositide 3-kinase (PI3K)/protein kinase B (AKT), *RAS/RAF*, and mitogen-activated protein kinase (MAPK) (Fig. 1), interleukin-6 (IL-6), vascular endothelial growth factor (VEGF), etc., which promote cancer aggressiveness [13,14,40]. HER2 alteration occurs in about 15%–30% of breast cancers and 10%–30% of gastric/gastroesophageal cancers, providing crucial predictive and prognostic markers. HER2 alterations are strongly associated with poorer prognosis in breast cancer [14,45–47]. In recent decades, numerous HER2-targeted agents, including small-molecule tyrosine kinase inhibitors (TKIs), have been developed and approved for the treatment of breast cancer. Afatinib was one of the first evaluated; the drug demonstrated promising anti-breast cancer effects in phase I and II clinical trials but failed in phase III [48–50]. However, tucatinib, pyrotinib, and lapatinib demonstrated better clinical outcomes in phase III [51–53]. HER2-targeted regimens remain an essential option for treating various HER2-altered cancers and may be considered potential targets for CCA treatment regimens in the future, including cases with moderate and low HER2 expression.

## 4 HER2 Alterations and Clinical Significance in CCA

In the past decade, numerous studies have indicated that Cholangiocarcinoma (CCA) exhibits significant heterogeneity and genomic alterations [15,26,54]. Notably, the genetic profiles of CCA depend on the underlying etiology, which is predominantly linked to infections caused by liver flukes, specifically *Opisthorchis viverrini* and *Clonorchis sinensis*, commonly found in Southeast Asia and China, respectively [54]. Jusakul et al. [15] demonstrated using comprehensive Omics analysis, copy number, and methylation profiles of 489 CCA cases (both non-fluke- and fluke-related CCAs) that CCA could be classified into four molecular subtypes. Clusters 1 and 2 are almost liver fluke-related CCAs, predominantly with *ERBB2* or *HER2* alterations, and enrichment with *TP53*, *ARID1A*, and *BRCA1/2* mutations. Clusters 3 and 4 demonstrated up-regulation of immune-related pathways such as *PD-1/PDL2*, *IDH1/2* mutations, and the fibroblast growth factor receptor (FGFR) family alteration, respectively. Moreover, fluke-related CCAs (cluster1/2) illustrated poorer survival compared with the fluke-negative subtypes. This suggests that chronic liver fluke infection could play a significant role in creating the differing epigenetic and genetic profiles observed between Asian and Western CCAs. Additionally, HER2 alterations may represent a distinctive feature of fluke-positive CCA. Current research indicates that alterations of HER2 may contribute to tumorigenesis in CCA, as demonstrated in transgenic mouse models. Persistent overexpression of HER2 in the basal layer of the biliary tract epithelium resulted in the formation of gallbladder adenocarcinoma in this mouse model [55]. Moreover, the overexpression of HER2 enhanced aggressive phenotypes of fluke-positive CCA cell lines, including proliferation and invasion/migration via PI3K/Akt pathways [56]. Recent *in vitro* research also reported that high glucose and lactic acidosis conditions promoted aggressiveness of fluke-positive CCA cell lines via the *EGFR/STAT3-*dependent and ALDH1A3/EGFR axis, respectively [57,58]. These perspectives may indicate that CCAs might be advancing through the EGFR axis. However, further studies are needed to confirm these viewpoints. Notably, the large-scale meta-analysis revealed that 26.5% of CCA patients are HER2 positive [59,60]. The immunohistochemical (IHC) analysis based on the site of the primary tumor revealed that HER2 was positive in 17.4% of eCCA, 4.8% of iCCA, and 19.1% of gallbladder cancer (GBC). Jusakul et al. demonstrated HER2 amplification was more frequent in liver fluke-related CCAs (10.4% of fluke-related CCAs vs. 2.7% of non-fluke CCAs) [15,61]. Chaisaingmongkol et al. found that *HER2* and ERBB3 are important oncogenic driver genes in Asian iCCA and hepatocellular carcinoma (HCC) [62]. HER2 amplifications and mutations in *MAP2K4*, *RASA1*, and *SF3B1* were reported as genetic drivers between iCCA and eCCA [15,26]. In a more recent study of surgically resected 454 CCA cases, HER2 was positive (IHC3+ or IHC2+ plus *HER2* gene amplification) in approximately 14.5% of all cases, including 18.5% of dCCA, 3.0% of pCCA, 3.7% of iCCA, and 31.3% of GBC. HER2 heterogeneity (defined as 5% or more of cancer cells exhibiting varying HER2 status) was observed in up to 83% of all cases. It correlated with the tumor histology of GBC, and a reduction in HER2 protein expression in the deeper invasive areas, along with simultaneous dedifferentiation, was commonly observed in HER2-positive cancer cells. The heterogeneity was not correlated with patient outcome; however, it’s important to carefully check HER2 levels using sensitive tests on larger pieces of the tumor. This will further aid in evaluating the impact of HER2 heterogeneity on HER2-targeted therapy in the near future [59,63]. Regarding prognostic significance, fluke-related CCAs (enriched with HER2 alterations) demonstrated poorer prognosis. Likewise, in Lowery et al.’s study, HER2-altered CCAs demonstrated significantly shorter progression-free survival (PFS) under first-line chemotherapy and a significantly lower median overall survival (mOS) compared with HER2 wild-type CCAs [59,64].

## 5 HER2-Specific Antibody Therapy

Trastuzumab, the humanized monoclonal antibody targeting the extracellular domain 4 (ECD4) of the HER2 receptor [52], was first approved as a therapeutic antibody in 1998 for HER2-positive breast cancer as a single agent or in combination with paclitaxel [65,66]. Since then, it has led to significant improvements in clinical outcomes in several metastatic diseases [67]. Trastuzumab illustrated direct anti-cancer activities through multiple mechanisms such as blocking receptor functions, regulating surface receptor expression and downstream signaling cascades leading to cell cycle arrest in the G1 phase, down-regulating cyclin E, suppressing tumor angiogenesis, impairing DNA repairing processes, which escalate susceptibility to conventional chemotherapy-induced apoptosis [65,68,69]. Not only does trastuzumab exert a direct anti-tumor effect, but it also exerts immunologic responses by facilitating antibody-dependent cell-mediated cytotoxicity (ADCC), the major function of natural killer cells against cancer [70,71]. Other studies demonstrated that macrophage-mediated antibody-dependent cellular phagocytosis (ADCP) is one of Trastuzumab’s mechanisms of action [72]. Recently, Panaampon et al. found that trastuzumab exerts multiple mechanisms against HER2-altered CCA, including direct growth inhibition, complement-dependent cytolysis (CDC), ADCC, and ADCP in both *in vitro* and CCA patient-derived xenograft (PDX) models [73].

The other HER2-specific monoclonal antibody is pertuzumab, which targets the HER2 extracellular domain 2 (ECD2). The antibody exerts an anti-tumor effect by tightly inhibiting the homodimerization of cytoplasmic tyrosine kinase regions of HER2 and heterodimerization of HER2/HER3, which are important steps for HER2-mediated oncogenic signaling cascades. The drug has been approved by the Food and Drug Administration and the European Medicines Agency for metastatic breast cancer [74,75]. As for combination regimens, it seems that trastuzumab plus pertuzumab or Phesgo, trastuzumab plus pertuzumab plus Hyaluronidase-zzxf, which acts as a dispersion agent to modify the permeability of human connective cancerous tissues, offer better clinical benefits [76]. The FDA approved the regimens in June 2020 for treating HER2-positive both early and metastatic breast cancers, while trastuzumab with tucatinib has been granted accelerated approval for colorectal cancer recently [76,77].

HER2-specific monoclonal antibodies (mAbs) demonstrated favorable clinical outcomes in HER2-positive breast cancer (IHC score 3+ or IHC2+ with gene amplification positive on an *in situ* hybridization (ISH)). However, HER2-targeted mAbs such as trastuzumab failed to provide clinical benefit in low-HER2-expressing (IHC2+ with ISH- or IHC1+) [78–80], which is reported to be observed in approximately 50% of breast cancer patients and other mass-forming types [81–83]. Some of the trastuzumab resistance mechanisms have been reported, such as overexpressing Akt, TGF-α, IGF1R, VEGF, etc. [84]. Not all tumors that overexpress HER2 exhibit a favorable response to these therapies; many that initially respond later acquire resistance. HER2-targeted drug-resistant cancer cells correlate with increased levels of immunosuppressive molecules such as transforming growth factor-beta 1 (TGFβ1) and programmed cell death ligand 1 (PD-L1), causing resistance to the anti-tumor immune response. Moreover, these molecules are encapsulated within extracellular vesicles (EVs), which facilitate the transfer of resistant phenotypes to drug-sensitive recipient cells. The levels of EV-associated TGFβ1 are also significantly correlated with the therapeutic response to HER2-targeted therapies in HER2-overexpressing cancer [85]. Moreover, other factors in the tumor microenvironment (TME), such as collagen, induce the expression of interleukin-4 induced 1 (IL4I1), contributing to T-cell exhaustion, and promoting immune evasion [86]. Additionally, Intratumor heterogeneity of HER2 expression/amplification within a single tumor present in breast cancer might affect treatment responses to HER2-targeted therapy, including trastuzumab, as the fraction of HER2 non-amplified cells remains and acts as a driver of therapeutic resistance [87]. Notably, the poor response rate and shorter survival of trastuzumab treatments were associated with overall low HER2 expression and intratumor heterogeneity [88,89]. Adverse effects of trastuzumab are associated with a decline in left ventricular ejection fraction (LVEF), which is linked to the expression of HER2 and ErbB4 receptors in cardiomyocytes [90]. In the pivotal clinical trial carried out by Slamon et al., trastuzumab plus anthracyclines, patients with advanced breast cancer experienced substantial clinical benefits; however, this combination resulted in an unacceptably high incidence of heart failure, occurring in 28% of cases [90–92]. With these limitations, we can move forward with the antibody-drug conjugate (ADC).

## 6 HER2-Specific Antibody-Drug-Conjugate (ADCs)

In the last decade, ADCs have gained popularity in cancer research as they provide an ideal anti-cancer drug delivery system, which significantly increases the maximum therapeutic effect against cancer and reduces undesirable side effects [93]. The ADCs are comprised of potent cytotoxic agents, cancer-specific mAbs, and an appropriate linker to connect the drug molecules. Nowadays, several ADCs have been approved for clinical use. More than 70 ADCs are being investigated in clinical trials now. For HER2-targeting ADCs, only trastuzumab emtansine and trastuzumab deruxtecan are approved ADCs for advanced HER2-expressing breast and gastric cancers. Some HER2-targeting ADCs are now evaluated in late clinical trials (phase III), including trastuzumab duocarmazine (SYD985, T-Duo), BAT8001, and disitamab vedotin (Table 1) [94–96] (https://clinicaltrials.gov/study/NCT03500380) (accessed on 10 June 2025). Trastuzumab dacarbazine (T-Duo) is a second-generation ADC comprising trastuzumab conjugated with a DNA alkylating agent, dacarbazine, with a drug-to-antibody ratio (DAR) of 2.4 to 2.8. The pre-clinical study revealed that a cleavable linker of T-Duo offers an anti-tumor activity to overcome trastuzumab emtansine (T-DM1)-resistant breast cancer in PDX models [97]. Importantly, T-Duo illustrated a favorable profile by significantly prolonging progression-free survival in phase III clinical trials (NCT03262935) and is now being further evaluated in the following steps [98].

**Table 1:** Key features of clinically approved or late clinical trials (Phase III) ADCs targeting HER2

Antibody-drug conjugates	Trastuzumab emtansine (T-DM1)	BAT8001	Trastuzumab deruxtecan (T-Dxd)	Trastuzumab duocarmazine (SYD985, T-Duo)	Disitamab vedotin (RC-48)
Anti HER2 Ab	Trastuzumab	Trastuzumab	Trastuzumab	Trastuzumab	Disitamab
linker	Non-cleavable (SMCC linker)	Non-cleavable (with stable amide bond)	Cleavable (GGFG linker)	Cleavable (VC linker)	Cleavable (VC linker)
Drug antibody ratio (DAR)	3.5:1	Unknown	8:1	2.4 to 2.8:1	4
Warhead (cytotoxic agents)	Mertansine (DM1)	Maytansine derivative	Exatecan derivative (Dxd)	Duocarmazine (Duo)	Monomethyl Auristatin E (MMAE)
Mechanisms	Inhibit spindle fiber assembly	Inhibit spindle fiber assembly	DNA topoisomerase I inhibitor	DNA minor groove-alkylating agents	Inhibit spindle fiber assembly
Bystander cell killing effect	No	No	Yes	Yes	Yes
Diffusible cytotoxic moiety	No	No	Yes	No	Yes
Efficacy on HER2-positive (homogeneous) cancers	Yes	Yes	Yes	Yes	Yes
Efficacy on HER2-low (heterogeneous) cancers	No	No	Yes	Yes	Yes
Drug status	FDA approved	Phase III	FDA approved	Phase III	Phase III
References	[61]	[62,63]	[60,64]	[58,59]	[55–57]

T-DM1 is the first HER2-targeted ADC approved by the FDA. The ADC consists of trastuzumab that incorporates a non-cleavable linker with emtansine (DM1), and the cytotoxic microtubule spindle fiber inhibitor with DAR 3.5 [99,100]. T-DM1 demonstrated a favorable anti-tumor effect against HER2 overexpressing breast cancer as an adjuvant, first- and second-line therapy in phase III clinical trials but, less or no effect in low HER2 expression. The main toxicities of T-DM1 are considered from the payload. Fatigue, nausea, thrombocytopenia, rising transaminases, and epistaxis are often reported in adverse events. Almost all of these events generally reported low grades except severe thrombocytopenia (≥grade 3) [99,101]. Thus, patients with cardiac dysfunctions should avoid or adjust appropriate doses or discontinue treatment. BAT8001 is one of the HER2 targeting ADCs (Table 1) comprising trastuzumab biosimilar (BAT0606) connected to a novel covalently uncleavable linker via a stable amide bond to avoid the release of toxic payloads in blood and increase stability to minimize toxicities. The antibody was conjugated with the maytansine derivative, the spindle fiber inhibitor. BAT8001 demonstrated anti-cancer activity by inhibiting the proliferation of HER2-expressing cells in both *in vitro* and *in vivo*. Moreover, no adverse effect level has been observed in cynomolgus monkeys. The drug is being evaluated in phase III clinical trials with a favorable safety profile similar to T-DM1 and demonstrates better multiple-dose toxicity than T-DM1 [102,103].

Trastuzumab deruxtecan (T-Dxd) is currently considered the most promising ADC against HER2-positive cancers with a maximum drug antibody ratio (DAR: 8.0). The trastuzumab was homogenously conjugated with 8 molecules of Topoisomerase I (TOP1) inhibitors using an enzymatically cleavable peptide-based linker (Table 1). The linker-drug of T-Dxd demonstrated stability in systemic circulation and selectively targeted HER2-expressing cancer cells by facilitating the antitumor effects of trastuzumab, including ADCC, ADCP, and CDC, and was selectively cleaved by intracellular lysosomal proteases such as cathepsins in cancer cells, allowing for the payload release. Moreover, the Dxd possesses high plasma membrane permeability to exert a potent bystander-killing effect against cancer heterogeneity and the tumor microenvironment [104,105]. T-Dxd not only exerts potent anti-tumor activity against high HER2-expressing cancer but also low-HER2-expressing and tumor microenvironment cells as well [106–108]. These might be an advantage of T-Dxd for fighting against HER2 intratumorally heterogeneities in CCA tumor mass and elimination of tumor microenvironment cells, such as cancer-associated fibroblasts (CAFs). Likewise, the most recent clinical trial (DESTINY-Breast03) demonstrated that T-DXd offers significantly longer progression-free survival than T-DM1 in HER2-altered breast cancer patients [109]. A systematic review revealed that the most common adverse events associated with T-DXd in all grades were nausea, fatigue, vomiting, anemia, constipation, neutropenia, and diarrhea. In terms of adverse events of grade 3 or more, only anemia and neutropenia occurred at a relatively high rate. The rate of nausea was 72.8% to 73%, anemia was 30.4% to 33.2%, while 42.8% of patients were treated with T-DXd, with 19.1% experiencing neutropenia of grade ≥ 3 [110]. Although T-DXd contains trastuzumab, the incidence of cardiotoxicity reported so far has been low. In the DESTINY-Breast01 trial, three patients experienced LVEF; two cases were grade 2 and one was grade 3, and all were asymptomatic; patients recovered or were recovering after therapy interruption. There were no events of cardiac failure associated with the LVEF decrease [92,111].

DHES0815A is another most recent clinically investigating (phase I trial: NCT03451162) HER2-targeted ADC. The humanized 7C2 (hu7C2) HER2-targeting antibody has been chosen as the core immunoglobulin for this antibody-drug conjugate (ADC) to enable a potential combination with current HER2 therapeutic antibodies. The hu7C2 recognizes explicitly the extracellular domain 1 (ECD1) of the HER2 receptor, distinguishing it from trastuzumab (ECD4) and pertuzumab (ECD2), which target different epitopes. Fluorescence-activated cell sorting (FACS) analysis revealed that hu 7C2 illustrated full binding to HER2-positive breast cancer cells in the presence of trastuzumab and pertuzumab. DHES0815A exerted promising anti-tumor activity against HER2-positive cancer cells (both direct- and bystander-killing effects) by the novel cleavable methyl-disulfide linker connected to DNA minor groove crosslinking agent, pyrrolo [2,1- c] [1,4] benzodiazepine monoamide (PBD-MA), the cytotoxic warheads with DAR: 2.0. For ADCC mechanism, trastuzumab illustrated slightly better than huC72, likely due to closer proximity of trastuzumab recognition site (ECD4) which, facilitating closer synapsis of immune effector and cancer cells [112]. The cytotoxic warhead, PBD-MA, demonstrated a reduction in cell viability of both highly proliferative and quiescent cancer cells in an *in vitro* study [112,113]. This might enhance the potency of the bystander effect against the acquiescent cancer cell population inside the tumor mass. Although DHES0815A has less payload (DAR: 2.0), it demonstrated more potency than T-DM1 (DAR: 3.5). Moreover, an overall preclinical animal model suggested equivalent anti-tumor efficacy between DHES0815A and T-Dxd. The most frequently reported adverse events of DHES0815A from phase I are pruritus, rash, phobia, hyperpigmentation of the skin, nausea, and fatigue [112,114,115]. Currently, several HER2-targeting ADCs are in early phase (I/II) development (Table 2).

**Table 2:** Novel ongoing early clinical study (phase I/II) of HER2 targeting ADCs on the horizon

ADCs	BL-M07D1	DHES0815A	Zanidatamab Zovodotin	ARX-788	ALT-P7
Anti HER2 Ab	Trastuzumab	hu7C2	Zanidatamab	modified trastuzumab	Trastuzumab
Linkers	Cleavable linker (Cathepsin B)	Cleavable methyl-disulfide linker	Cleavable linker	Non-cleavable	Cleavable cysteine peptide
Drug antibody ratio (DAR)	8	2	2–4	1.9	2
Warhead (cytotoxic agents)	Ed-04	PBD-MA	Zovodotin	MMAF (AS269)	MMAE
Clinical trial phase	Phase I	Phase I	Phase I	Phase II	Phase II
References	[116,117]	[112]	[118–120]	[115]	[121,122]

Notes: humanized 7C2, hu7C2; amberstatin-269, AS269; Edotecarin-04, Ed-04; pyrrolobenzodiazepine-monoamide, PBD-MA; Monomethylauristatin F, MMAF; Monomethyl auristatin E, MMAE.

Together, HER2-targeting ADCs represent a promising platform for cancer immunotherapy [123]. They demonstrate anti-cancer effects through several mechanisms, including the typical responses linked to the fragment crystallizable (FC) portion of mAbs, such as ADCC, ADCP, and CDC, in addition to their specific cytotoxic payload mechanisms. Advanced linker technology offers various strategies for anti-cancer drug delivery systems to combat HER2-altered cancer, including CCA, while minimizing the unintended release of payloads.

## 7 HER2 Targets Antibodies, Affibodies, and Nanobodies for Radioimmunotherapy

Not only for precisely delivering cytotoxic agents to cancer cells but mAbs are also applied in cancer theranostics (radionuclide imaging and radiation therapy) as vehicles for radionuclide conjugates. For example, Lutetium-177 (Lu-177)-trastuzumab, which is radiolabeled trastuzumab with a bifunctional chelator, dodecane tetraacetic acid (DOTA), and Lu-177, a medical isotope in targeted radionuclide therapy for treating neuroendocrine- and prostate malignancies [124]. The radioimmunoassay revealed good antigen-binding affinity and specificity of Lu-177-trastuzumab against the HER2 cell surface receptor. Moreover, the patient’s studies illustrated the localization of Lu-177-trastuzumab at both primary and metastatic sites of HER2-positive breast cancer using the combination of Single Photon Emission Computed Tomography scan and Computed Tomography (SPECT/CT) imaging [124]. However, no tumor uptake was detected in HER2-negative patients, indicating the specificity of Lu-177-trastuzumab for HER2-positive cancer diagnosis and probably becoming targeted radionuclide therapy for HER2-positive cancer shortly. Another interesting strategy is near-infrared photoimmunotherapy (NIR-PIT), which is the application of mAbs for carrying the inactive form of photo-absorbing chemical cytotoxic agents to cancer cells. Once the conjugated antibodies bind to cancer cells, near-infrared light is locally provided to activate the photo-absorbing chemical molecules, which results in direct damage to cancer cells and minimizes damage to adjacent normal cells [125,126]. The cell death via NIR-PIT demonstrated distinct characteristics as necrosis, which forms cell swelling, membrane blebbing, and membrane disruption, resulting in vigorous anti-tumor immune responses. IRDye^®^ 700 is one of the most popular photo-absorbing molecules. Sato and the team conducted the dual targeting of EGFR and fibroblast activation protein (FAP) by conjugation of panitumumab, the EGFR targeting antibody, and anti-FAP with IRDye700. The results revealed that dual-targeted EGFR/FAP NIR-PIT significantly offers a therapeutic effect against cancer cells and CAFs simultaneously *in vitro* and *in vivo* mice models [127].

Monoclonal antibodies such as trastuzumab or pertuzumab are often used as vehicles for precisely delivering cytotoxic agents or radionuclides to HER2-expressing cancer cells for therapeutic and imaging diagnosis. Although ADCs demonstrated promising clinical profiles against HER2-altered tumors, there are still some insurmountable limitations for mAbs such as heterogenous blood perfusion profiles, slow blood clearance which is also associated with tumor uptake, tumor penetration since the molecular weight of antibodies is relatively high (approximately 160 kDa), extravascular binding resulting in increasing turgor effect (interstitial pressure) leading to heterogenous intratumor distribution or stuck at the periphery of the tumor mass. The relatively large molecular size of mAbs makes them unable to be filtered by the kidney glomerulus and accumulation in the liver, leading to nephrotoxicity or hepatotoxicity. In addition, few therapeutic antibodies can pass through the blood-brain barrier, making it difficult for brain metastasis treatment. Together, these drawbacks initiated further development of smaller molecules of antibody fragments to improve therapeutic efficacy and molecular imaging of cancers by radiolabeled [128,129].

Affibodies are engineered from small antibody fragment molecules that could be applied for theranostics. Affibodies derived from three cysteine-free helical sub-domain scaffolds protein (58 amino acids in length) without disulfide bridges, which cause nonspecific binding to various proteins with a high affinity [130]. Due to their small size, quicker blood clearance, and high affinity, they are attractive for molecular imaging. The first clinical trial of molecular imaging for HER2-positive breast cancer using radiolabeled HER2-targeting affibody ABY-002 was promising. High-quality positron emission tomography (PET) and single-photon emission computed tomography (SPECT) results were acquired 2 h after injection (p.i.) with gallium-68- and indium-11-labeled ABY-003, respectively [131]. Currently, this tracer has been further developed to achieve a better blood clearance profile and reduce image background. Although affibody demonstrates several advantages and properties that make it useful, particularly for molecular imaging, some hurdles must be overcome. The labeling process can lead to increased lipophilicity of the molecule, which might lead to non-specific interaction with normal tissue, as well as the development of affibody, nowadays still expensive [129]. A further important disadvantage is that affibody could be the origin of bacterial scaffold proteins, which risks increasing the immunogenicity of patients after several administrations [132]. Further study is required to improve therapeutic application, which is hampered by the short retention time of the affibody in blood.

Nanobodies are derived from the variable domain of the heavy chain of heavy-chain antibodies (VHH). Heavy chain antibodies (HcAbs) represent a class of antibodies that consist exclusively of two heavy chains and do not contain light chains. HcAbs could be found in camelid species such as *Camelus dromedarius*, *Camelus bactrianus*, *Lama glama*, and sharks [133,134]. Since the lack of light chains, HcAb binds to the antigen by only a single variable domain connected to the FC portion (CH2 and CH3) by a hinge domain. The VHH of HcAb offers a functional equivalent to the fragment antigen-binding region (Fab) of traditional antibodies. Nanobodies provide several advantages to overcome conventional antibodies, such as high affinity, stable molecules, high water solubility, small sizes of 2.5 and 4.0 nm for width and length, with a molecular weight of approximately 15 kDa. Due to their small size, nanobodies are rapidly cleared by the kidneys, resulting in a short biological half-life. Additionally, nanobodies share a high degree of sequence homology with human VH domains, which contributes to their low immunogenicity [128,135]. These properties make nanobodies suitable for tumor tissue penetration and specifically bind to cancer antigens. In the manner of the theranostics tool, nanobodies should be tagged with a short physical half-life of radionuclides. The first clinical study with radioactively labeled nanobody for PET/CT was conducted by Keyaerts and team [136] ^68^Ga-NOTA-2Rs15d. The favorable biodistribution of the tracer was obtained as accumulation in HER2-positive breast cancer metastases is high, with a very low background compared with normal adjacent tissues. In addition to their use in cancer diagnostics, recent studies have increasingly explored the therapeutic potential of nanobodies. Several therapeutic nanobodies, labeled with various radionuclides, have shown promising anti-cancer effects. For example, ^131^I-SGMIB-2Rs15d as a single agent or in combination with trastuzumab demonstrated significantly improved median survival in animal models [137]. Interestingly, the study of ^225^AcDOTA-2Rs15d nanobody alone or in combination with trastuzumab offers prolonged median survival of brain metastatic breast cancer mice [138]. In contrast to conventional antibodies, radiolabeled nanobodies can pass through the blood-brain barrier, offering better options for molecular imaging of brain metastatic lesions and potentially serving as therapeutic agents.

## 8 Multi-Specific Cell Engager Molecules

Cell engager molecules are the modified antibodies with at least one arm targeting tumor-specific associated antigens. In contrast, other arms engage in activating domains of the immune effector cells, such as CD3 for T-cell recruitment or targeting the low-affinity immunoglobulin gamma Fc region (FcγRIII) known as CD16 for natural killer (NK) cell engagement. A decade before, the first bispecific antibody targeting CD3 and CD19 was first approved by the US FDA in 2014. Since then, gaining momentum of engager molecules in the cancer immunotherapy field until 2022, three Bispecific T-cell engagers were approved later on, four were approved in 2023, and one was just approved in 2024, respectively. T-cell engager technologies are now likely to revolutionize cancer immunotherapy, especially in hematological malignancies, as they offer more potent antitumor activities than classical monoclonal antibody therapies. Several current perspectives of cell engagers have been developed [139], such as bifunctional checkpoint-inhibitory T-cell engagers (CiTEs) [140], Simultaneous multiple interaction T-cell engaging (SMITEs) [141], dual-affinity re-targeting (DART) [142], Trispecific T-cell engagers (TriTE) [143], Trispecific Killer Engagers (TriKE) [144], etc., as summarized in Table 3. More than 100 bispecific T-cell engagers are being investigated in clinical trials [145,146]. Bispecific NK cell engagers are now being examined in the early phase of clinical trials. Furthermore, another binding arm may target additional tumor-specific antigens to increase cancer-specific binding and prevent cancer immune escape or target additional co-stimulatory immune effector cells. The tri-specific engagers were developed.

**Table 3:** Current and future perspectives of cell-engaging molecules

Cell engagers	Bifunctional checkpoint-inhibitory T-cell engagers (CiTEs)	Simultaneous multiple interaction BiTEs (SMITEs)	Dual affinity re-targeting (DART)	Trispecific T-cell engagers (TriTE)	Trispecific killer engagers (TriKE)
Targets (example)	PD-L1/CD3	CD3/TAA, and PD-L1/CD28	CD3/TAA	CD3/CD28-HER2	CD16/IL-15-CD33
Strategies	Combine the inhibitory effects of local immune checkpoints, the PD1-PD-L1 axis, with the redirection of T-cell functions in one molecule.	Consists of two separate BiTEs, including, 1. Convert PD-L1 inhibitory axis to positive signal via targeting PD-L1 and co-stimulatory of T cells, CD28. 2. Conventional BiTE, targeting CD3 and TAA.	Bispecific diabody comprising two Fv chains offers dual antigen binding domains assembled via disulfide bonding.	Enhancing T-cell activation via CD3 and CD28 against HER2-positive malignancy.	Armed NK-cell activities targeting IL-15 and CD16 against specific TAA (CD33).
Advantages	Tackle PD1/PD-L1 axis resistant mechanisms, limiting the risk of off-target immune-related AEs.	Further, enhances conventional BiTE by converting the inhibitory signal of the PD-1-PD-L1 axis to an activating signal.	Greater stability and optimal, biological activation of T-cells against malignant than conventional BiTE.	Further, enhance T-cell activities via CD28 against HER2 positive cancer cell.	Reduce cytokine release syndrome risk, swiftly kill cancer cells via NK-cells activation.
References	[139,140]	[141]	[142]	[143]	[144]

Cell engager technology offers a promising novel platform for cancer immunotherapy, especially for hematological malignancies. Currently, there are increasing trends for the application in mass-forming cancers by focusing on HER2 or HER3 including zanidatamab, the biparatopic HER2 targeting antibody, and zenocutuzumab (Zeno) which exerts anti-cancer activity by targeting HER2 and HER3 respectively. Moreover, targeting HER2 and CD3 in mass forming such as breast cancer using tri-specific cell engager molecules has been developed by adding further CD28 into another binding arm for co-stimulating T-cell response against HER2-positive breast cancer. The HER2 tri-specific antibody illustrated multiple anti-cancer effects such as T-cell stimulation, and activation of CD8 cytolytic functions, furthermore, they highlighted the importance of CD4-mediated tumor regression in humanized mouse models through the induction of breast cancer cell cycle arrest [143]. The tri-specific antibody also demonstrated favorable pharmacokinetic profiles in non-human primates animal models [143].

Zanidatamab has a special biparatopic structure (bispecific antibody) targeting 2 distinct HER2 epitopes including ECD2 and ECD4. These unique characteristics allow antibodies to bind to HER2 molecules in trans and initiate HER2 reorganization. Zanidatamab exerts several mechanisms of action, such as inhibiting intracellular signaling, ADCC, and ADCP, but strongly induces complement-dependent cytotoxicity against HER2-positive breast, gastric, esophageal, and lung cancer. This functionality is not observed with trastuzumab, pertuzumab, or a combination of both [147]. For *in vivo* xenograft and PDX models, zanidatamab illustrated better antitumor activity than the combination of trastuzumab and pertuzumab [147]. However, the mechanisms of CCA cells need further study. The antibody is now being evaluated and provides clinical benefit in the clinical trial phase 2 for HER2-altered CCA patients [118,148]. Zanidatamab has shown significant clinical benefits while maintaining a manageable safety profile against HER2-amplified CCA patients. With an ORR of 41% and 68% of disease control rate (DCR). A total of 29 out of 87 participants (33%) experienced side effects such as chills, fever, or high blood pressure. There were no grade 4 treatment-related adverse events and no treatment-related deaths [118].

Zeno, the bispecific humanized antibody (IgG1), contains two different Fabs that target the extracellular domain of HER2 and HER3. The binding against HER3 prevents *NRG1* from binding to HER3 in order to prohibit heterodimerization with HER2 [149–151]. Zeno illustrated growth inhibition by cell cycle arrest as well as induction of ADCC against *NRG1-*altered breast and lung cancer cell lines in preclinical *in vitro*, *SLC3A2-NRG1* and *CD74*-*NRG1* gene fusions lung cancer patient-derived xenograft models (PDXs) showed suppressed growth inhibition and tumor regression. The Zeno is now being FDA-reviewed for use in Neuregulin 1 fusion (NRG1^+^) non-small cell lung cancer (NSCLC) and NRG1^+^ pancreatic cancers (PDAC).

Cell engager strategies may offer advantages over traditional therapeutic antibodies, including better tumor penetration because of their smaller size. They can also target multiple tumor-associated antigens, which may improve specificity and prevent the shedding of tumor antigens. Additionally, they may allow for the engagement of co-stimulatory domains of immune effector cells, potentially enhancing the immune response to cancer cells. Targeting HER2 along with other tumor surface molecules, such as FGFR or PD-L1, using multi-specific cell engagers may serve as a viable therapeutic option for HER2-altered CCA shortly.

## 9 CAR Immune Cells

Adoptive T-cell therapies (ACT) have become one of the pillars of cancer immunotherapy since the PD1/PD-L1 axis was discovered. Three platforms for ACTs currently exist: first, using isolated tumor-specific T-cells from (tumor-infiltrating lymphocytes; TIL) of patients’ samples, which are *ex vivo* expanded and therapeutically reinfused. Secondly, isolated T-cells from peripheral blood mononuclear cells (PBMCs) were genetically modified to express specific T-cell receptors (TCRs) that target cancer-specific antigens [152]. Third, T-cell modification using a fully synthetic chimeric antigen receptor (CAR) [153,154] with specific characteristics to recognize the desired tumor antigen. Unlike the physiological TCRs, CARs could recognize cancer antigens regardless of the major histocompatibility complex (MHC). This particular advantage appears when MHC expression is lost due to the cancer’s immune evasion mechanism. CAR consists of three primary components: the extracellular domain, which is primarily for recognizing tumor-specific antigens; the hydrophobic transmembrane domain; and the intracellular domain (endodomain) responsible for downstream signal transduction following antigen binding. The cytoplasmic T cell co-receptor, T-cell surface glycoprotein CD3 zeta chain (CD3ζ), contains three immunoreceptor tyrosine-based activation motifs (ITAMs), which are essential components for most CARs [155].

Nowadays, five generations of CAR T-cells have been created based on the composition of the cytoplasmic domains. The first generation of CAR contains only a single CD3ζ region, which lacks co-stimulatory domains such as CD28, CD27, and CD134, and cytokines, such as IL-2 signaling [155,156]. As a result, the first generation of CAR demonstrated low proliferation and cytotoxicity in the initial experiments. The second generation of CAR included co-stimulating parts such as CD28 or CD137 in the cytoplasmic domain to enhance proliferation and activate cytotoxic killing of T-cells [157,158]. The third generation was developed by further extending the co-stimulatory domain from the second generation, including CD134 and CD137. Additionally, the incorporation of cytokine production, specifically interleukin 12 (IL-12), which is consistently expressed upon CAR activation, was designed for the fourth generation, often referred to as T cells redirected for universal cytokine-mediated killing (TRUCK). The activation of this CAR construct offers a tumor-killing effect via multiple synergistic mechanisms such as perforin, granzyme exocytosis, and death ligand-death receptors (Fas-FasL, TRAIL) [156,159]. The fifth generation is now being established and evaluated. The fifth construct is also based on a second-generation construct but comprises a truncated cytoplasmic IL-2 receptor beta chain with a STAT3 binding site. Of this strategy, once tumor antigen activation of the receptors simultaneously with TCR (through CD3ζ regions) along with CD28, the co-stimulatory regions, as well as JAK-STAT3/5 cytokine signaling, fully drive T-cell proliferation and activation [160].

Since the first CAR-T cell was developed, it has demonstrated promising profiles in several clinical trials against CD19-positive hematological malignancies such as acute B-cell lymphoblastic leukemia with unprecedented clinical benefits of up to 81% [161–163]. Now, the FDA has approved six CAR-T cell therapies for clinical use [164–166]. Since CAR-T cell therapies offer an excellent clinical profile against hematological malignancies. Up to 80% ORR and patients are going into complete remission with minimal side effects [167–169]. Nowadays, several CARs have been modified to fight against solid mass-forming malignant antigens such as disialoganglioside (GD2), L1 cell adhesion molecule (CD171), folate-binding receptors, HER2/ERBB2, and vascular endothelial growth factor receptors 2 (VEGFR2) [170]. In contrast to blood cancers, CAR-T cell therapies failed to provide significant clinical benefits in solid tumors and led to severe or lethal side effects reported in several attempts [171–173]. Several challenges have to be considered, such as intra-tumor heterogeneity (ITH), hypoxic conditions, physical barriers, poor vascularization, shedding/loss of tumor antigens, tumor microenvironments, infiltrating T-cells, etc. Together with these reasons, targeting mass-forming cancers with CAR T-cell therapies still has many obstacles to be concerned with. For example, increasing tumor recognition of T-cells, preventing T-cell exhaustion, and increasing tumor-mass infiltration of T-cells. Various strategies have been applied to enhance T-cell tumor recognition and activation, such as CAR T cells secreting cell-engaging molecules, and the cancer vaccine with the mechanisms to emphasize antigen presentation of various solid tumor antigens, enabling protective immunity against cancers [154].

## 10 The Clinical Trials of HER2 Targeting Immunotherapy in HER2 Altered CCAs

While there isn’t a specific standard just for CCA, the scoring system used for IHC, based on criteria from breast and stomach cancers, defines HER2-positive results as either an IHC score of 3+ or an IHC score of 2+ combined with a positive result from ISH [174]. However, next-generation sequencing (NGS) is recommended for advanced CCA patients. In case of insufficient tumor biopsy samples, circulating tumor DNA (ctDNA) from patients could be used as an alternative way, with an 80% predictive value for HER2 positivity [175]. Recently, several clinical trials demonstrated the benefit of HER2 targeting for immunotherapy in HER2-altered-CCA patients (Table 4). For instance, trastuzumab in combination with tucatinib, a kinase inhibitor, demonstrated a favorable ORR: 46.7% [176]. Moreover, in the KCSG-HB19-19 trial, trastuzumab with FLOFOX regimen (leucovorin, 5-FU, and oxaliplatin) illustrated a favorable disease control rate of 79.4%, however, a lower ORR: 29.4% in 34 patients with HER2-positive CCA (IHC3+ or 2+ with ISH amplification) [177]. Zanidatamab, the biparatotic targeting HER2 antibody, revealed meaningful activity in clinical trials against HER2-positive CCA (IHC3+ or 2+ and ISH+), such a rapid and durable response, safety profile with ORR: 41%, disease control rate (DCR) 68.8% and duration of response (DoR) 12.9 months [118]. Interestingly, blocking the interaction of PD-L1/PD1 using the human immunoglobulin G1 kappa (IgG1κ) monoclonal antibody, durvalumab plus gemcitabine and cisplatin as first-line therapy, demonstrated a shorter overall survival trend in the ERBB2 altered CCA subtype. Supporting the evidence that no clinical benefit to including durvalumab in the strategy [178].

**Table 4:** Clinical trials of HER2 targeting immunotherapies in advanced CCA

No.	Trials (names)	CCA subtypes included and patients’ characteristics	Overall response rate (ORR)	Disease control rate (DCR)	% grade ≥3 AEs	Ref.
1.	Trastuzumab + Pertuzumab (Mypathway)	39 CCA patients (IHCC, EHCC, gallbladder) with IHC3+ and/or amplification via (HER2:CEP17 ratio >2 or HER2 copy number > 6) or NSG upregulated	23.0%	51.0%	46.0%	[179]
2.	Zanidatamab (HERIZON-BTC-01)	80 CCA patients (IHCC, EHCC, gallbladder) with IHC3+ or 2+ and ISH+	41.0%	68.8%	18.0% (only grade 3)	[118]
3.	Trastuzumab + Tucatinib (SGNTUC-019)	30 CCA patients with IHC3+ or amplification via ISH or NGS	46.7%	76.7%	60.0%	[176]
4.	Trastuzumab + FOLFOX (KCSG-HB19-14)	34 CCA patients (IHCC, EHCC, gallbladder) with IHC3+ or 2+ and ERBB2 amplification via ISH or gene copy number ≥ 6 of NGS	29.4%	79.4%	85.3%	[177]
5.	Trastuzumab Deruxtecan (HERB)	32 CCA patients (IHCC, EHCC, gallbladder, ampullary) with both HER2 positive (IHC3+ or IHC2+/ISH+) HER3-low (IHC2+/ISH-, IHC1+/ISH+, or IHC0/ISH+	36.4% (HER2 positive) 12.5% (HER2-low)	81.8% 75.0%	81.3%	[180]
6.	Trastuzumab Deruxtecan (DESTINY-PanTumor02)	41 CCA patients with (IHCC, EHCC, gallbladder, ampullary)	22.0%	78.0%	39.0%	[181]

In addition, HER2-targeted ADCs such as T-Dxd exert antitumor activities against a broad range HER2 HER2-expressing breast and gastric cancers in clinical trials and clinical use. Likewise, in CCA, T-Dxd achieved disease control rates up to 75.0% and 81.8% of HER2-low and HER2-positive CCA patients, respectively [180]. Moreover, 36.4% of ORR and 7.4 months of DoR have been observed in 8 HER2-positive CCA patients (five patients for IHC3^+^, three patients for IHC2^+^/ISH^+^ patients, and one HER2-low) [180]. A similar trend has been reported in the DESTINY-panTumor02 clinical trial showed that T-Dxd offers 56.3% ORR in 16 IHC3^+^ CCA patients [181]. This might be evidenced that T-Dxd possesses potent anti-tumor activity across subtypes and a broad range of HER2-expressing CCAs. However, the mechanisms underlying T-Dxd against CCA have not been fully explored and urgently need further preclinical studies supporting this point of view. Together, these suggest the potential application of specific HER2 targeting immunotherapy for HER2-altered CCA clusters with favorable clinical outcomes and emphasize further developing alternative treatment strategies for CCA patients [118,179,182].

## 11 The Application of Mouse Models in Preclinical Cancer Therapy

Nowadays, it is undeniable that multi-omics studies have revealed several novel potential therapeutic target molecules for cancer therapy, including CCA. However, only the studies from several *in vitro* models are insufficient to recapitulate the cancer’s pathophysiological environment and complexity in the human body, as evidenced by the substantial failure rate of compounds during the drug development and discovery processes. To unravel the complexities inherent in cancer, mouse models have become a pivotal bridge between *in vitro* evaluation and clinical practice. The development of preclinical animal mouse models allows the researcher to understand the multidimensionality of various crucial aspects of cancer. Rodentia mainly comprises mice, rats, and guinea pigs, which are at the forefront of the preclinical animal models, particularly anti-cancer drug developments. Four are prevalently employed for cancer studies, including Syngeneic and Xenotransplantation, *in situ* model, genetically engineered, and PDXs.

1.) Syngeneic and Xenotransplantation models: Syngeneic transplant, also known as the allograft mouse models, refers to the use of immunodeficient mice as recipients of cancerous cells derived from mice or genetically similar rodents. While the xenograft models refer to the use of immunodeficient mice as recipients of human cancerous cells or primary tumor tissue from patients (transplant cells or tissues between different species) [183,184]. Each transplantation has its advantages and disadvantages. Syngeneic models provide rapid and reproducible tumor growth, which is easy to manipulate. However, it may not fully recapitulate the complexity of human tumors and demonstrate immune phenotypic differences [185]. In addition, the species available for syngeneic models are limited. Xenotransplantation offers various advantages among animal models and human cancers and more accurately reflects original human tumor architecture and microenvironments. However, the mismatch between tumor and host-microenvironment in different species might alter the molecular, immune, and cellular differences. This hinders this model and is also considered one of its weak points.

2.) *In situ* model: This is the *in vivo* tumor model that involves the induction of tumorigenesis in mice at the specific original site [186]. These offer more realistic tumor development and progressions, such as local invasion, and distal metastatic spread of the primary site, than the classical Syngeneic models [187]. The methods mainly include the induction of tumor initiation using ultimate chemical carcinogens or utilizing gene editing technology such as CRISPR to induce malignant transformation in mice through knock-out tumor suppressor genes or overexpressing oncogenes. However, one of the seemingly obvious disadvantages is that chemical carcinogens such as Dimethyl-Benz(a)anthracene (DMBA) and Azoxymethane (AOM) have toxic effects on normal adjacent tissue, and gene editing techniques are still relatively costly, complex, and limited in success rates.

3.) Genetically engineered mouse models: refer to mouse models that are established by editing key regulatory genes that relate to human tumorigenesis at the mouse’s genome level to induce spontaneous cancer initiation and pathological progression. This model provides a systemic investigation of the potent oncogenes or tumor suppressor genes, as well as studies the association of targeted anti-cancer drugs and related genes.

4.) Patient-derived xenograft models (PDXs): Xenotransplantation models using *in vitro* human cancer cell lines in mouse models are not always representative of the fundamental pathophysiology of human tumors. As for cancer cell line transplantation in mice often lacks tumor heterogeneity, intertumoral microenvironment, tumor-related matrix interaction, etc. Cancer cell lines somehow lost their original features of the original cancerous tissues after transplantation [188]. These might contribute to the distinct drug response and make it difficult to predict drug response at the clinical level or lack clinical relevance. Of these, PDX models, which refer to the transplantation of fresh biopsy tumor samples from a patient into immunodeficient mice, could overcome these drawbacks as they preserve the original tumor architecture and tumor microenvironment of the patient’s cancer tissue. PDX models illustrate extreme clinical relevance and predictive value for the translation of preclinical to clinical level. Moreover, this model also offers the potential in sign into treatment efficacy for precision medicine [189]. Nowadays, several immunodeficient mice have been developed and are available for PDX models. For example, NOD/SCID (NOD.Cg-Prkdc^scid^), which lacks mature B- and T-cells, and has impairment of NK-cells and macrophage functions [190], NSG (NOD.Cg-*Prkdc*^*scid*^*Il2rg*^*tm1Wjl*^/SzJ), which is one of the most widely used mice as it offers a severe immunodeficient phenotype due to no mature B-, T-cells, no NK-cells, and impaired macrophage/dendritic functions [191], and BALB/c Rag2-/-Jak3-/- (BRJ), which is characterized by a lack of mature B- and T-cells as well as NK-cells [192]. Interestingly, BRJ mice have been used as alternative recipients of CCA in both PDXs and CCA cell lines, showing a higher engraftment rate of CCA PDXs up to 75% [193]. Nowadays, BRJ mice have gained popularity in CCA preclinical research in both testing small-molecule inhibitors [194] and immunotherapy [73] as they offer not only a higher success rate of CCA transplantation but also a quality preservation of the original characteristic of the tumor, and the price is relatively lower than other strains.

The PDX platform illustrates a robust preclinical model that bridges preclinical and clinical evaluation. Nevertheless, the PDX model still requires further optimization and development. Human stromal components are still rapidly replaced with murine stromal cells during engraftment and passage of PDX [195]. The immune humanized cancer PDX model (firstly transplanted with human hematopoietic stem cells CD34+ for reconstitution of the human immune cells and followed by PDX transplantation) might be a better relevant model for studying the cancer-related immune response in immunotherapy. Further study is required to improve the PDX model as one of the most promising preclinical platforms.

## 12 Conclusions

**Figure 2:**
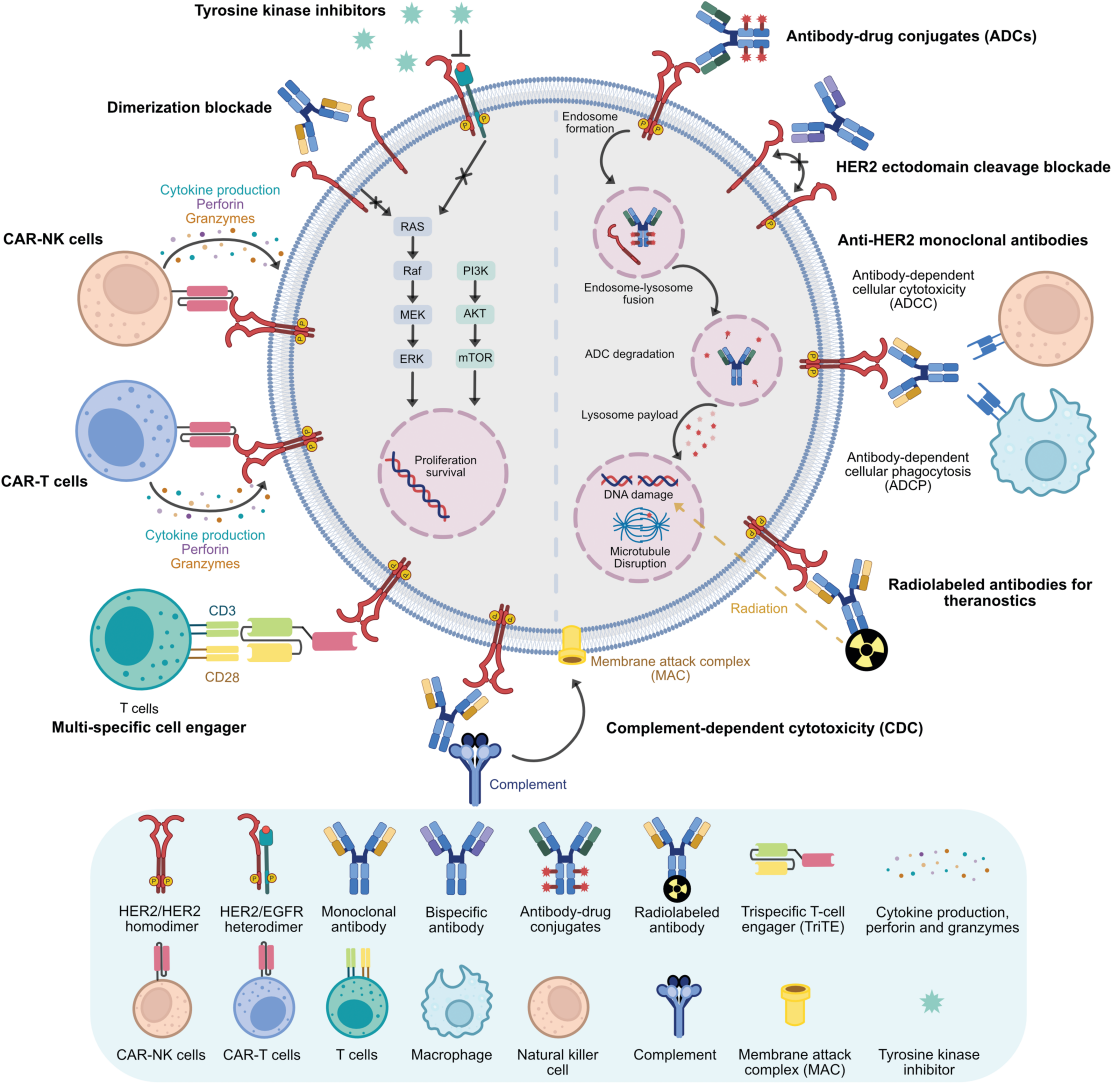
HER2 targeting immunotherapy platforms Overview of HER2 targeting strategies for immunotherapy against CCA (Created with Affinity Designer 2 software)

These insights highlight potential future directions for CCA immunotherapy, focusing on HER2 alterations. As demonstrated in the previous literature, HER2 alteration is dominant in the liver-fluke CCA (most of the Asian CCAs) as well as some small fraction of the Western world’s CCAs, and HER2 alterations are also significantly associated with inferior prognosis of CCA patients. Thus, targeting HER2-altered CCA is urgently needed. Currently, several strategies have been developed for HER2-altered cancers (Fig. 2) such as ADCs which precisely deliver cytotoxic warheads to kill cancer cells directly as well as exert bystander-killing effect against tumor microenvironment cells. Moreover, ADCs such as T-Dxd also possessed the anti-tumor mechanisms of conventional trastuzumab. Not only cytotoxic drug delivery systems but mAbs such as trastuzumab and pertuzumab are now applied for cancer theranostics in radioimmunotherapy as well. Although modification of whole mAbs demonstrated promising clinical profiles against HER2-positive tumors. However, there are still some insurmountable limitations of mAbs such as a more extended blood clearance period, inability to pass through the blood-brain barrier, and the tumor’s physical barrier, etc. These drawbacks minimize the therapeutic efficacy of mAbs against cancer, such as brain metastatic disease, or cause undesirable outcomes, such as nephrotoxicity and hepatotoxicity. Affibodies and nanobodies, which demonstrate quicker blood clearance and better tumor penetration due to their relatively smaller size, have been further developed to address these limitations. Both preclinical and clinical data showed quicker blood clearance and better blood-brain barrier penetration of radiolabeled HER2-targeted nanobodies in molecular imaging of brain metastatic lesions of HER2-positive breast cancer. This might be possible to apply in CCA, not only for molecular imaging but also for therapeutic options, such as nanobody drug conjugate, which should be considered for further study. Raising further with multi-specific cell engager strategies that revolutionize monoclonal antibody therapy by at least one arm targeting tumor-associated antigens, and one or two arms targeting co-stimulatory receptors of immune effector cells, or targeting additional tumor-specific antigens to increase cancer-specific binding and prevent cancer immune escape. Many engaging molecules exert multiple anti-cancer mechanisms in both preclinical and clinical studies. However, further investigation is necessary to avoid or minimize undesirable side effects such as cytokine release syndrome (CRS) as well as the development of CAR cells secreting engager molecules against HER2-altered CCA. In non-fluke-associated cholangiocarcinoma (most Western CCAs), molecular classification has identified specific clusters enriched with *PD-1*, *PD-L1*, and *PD-L2* expression signatures [15,54,61], which may derive more significant clinical benefit from durvalumab in combination with gemcitabine and cisplatin [34]. We also provided strong evidence from the current stage of literature about clinical trials of targeting HER2-altered CCA using several strategies of immunotherapy that provide favorable clinical outcomes and emerging HER2 as the potential target for CCA immunotherapy shortly.

## Data Availability

The datasets generated and/or analyzed during the current study are available from the corresponding author on reasonable request.

## Abbreviations

ACT: Adoptive T-cell therapies
ADC: Antibody-drug-conjugate
ADCC: Antibody-dependent cell-mediated cytotoxicity
ADCP: Antibody-dependent cellular phagocytosis
AOM: Azoxymethane
CAFs: Cancer-associated fibroblasts
CAR: Chimeric antigen receptor
CCA: Cholangiocarcinoma
CDC: Complement-dependent cytolysis
CiTEs: Bifunctional checkpoint-inhibitory T-cell engagers
CRS: Cytokine release syndrome
ctDNA: Circulating tumor DNA
DAR: Drug antibody ratio
DART: Dual affinity re-targeting
DCR: Disease control rate
DMBA: Dimethyl-Benz(a)anthracene
DoR: Duration of response
ECD2: Extracellular domain 2
ECD4: Extracellular domain 4
Fab: Fragment antibody binding region
FAP: Fibroblast activation protein
FDA: Food and Drug Administration
FGFR: Fibroblast growth factor receptor
5-FU: 5-fluorouracil
HcAb: Heavy chain antibodies
HER2: Human epidermal growth factor receptor 2
ISH: *In-situ* hybridization
ITH: Intra-tumor heterogeneity
NGS: Next-generation sequencing
NIR-PIT: Near-infrared photoimmunotherapy
NSCLC: Non-small cell lung cancer
ORR: Overall response rate
PBD-MA: Pyrrolo [2,1- c][1,4]benzodiazepine monoamide
PDX: Patient-derived xenograft
SE-Asia: Southeast Asia countries
SMITEs: Simultaneous multiple interaction BiTEs
SPECT/CT: Single Photon Emission Computed Tomography scan and Computed Tomography
TAA: Tumor-associated antigens
TCR: T-cell receptors
T-DM1: Trastuzumab emtansine
T-Dxd: Trastuzumab deruxtecan
TIL: Tumor-infiltrating lymphocytes
TKIs: Tyrosine kinase inhibitors
TOP1: Topoisomerase I
TriKE: Trispecific Killer Engagers
TriTE: Trispecific T-cell engagers
VEGFR2: Vascular endothelial growth factor receptor 2
